# Necrotizing scleritis after strabismus surgery in Treacher Collins syndrome

**DOI:** 10.3205/oc000147

**Published:** 2020-04-03

**Authors:** Soveeta Rath, Suma Ganesh, Umang Mathur, Manasvini Sharma

**Affiliations:** 1 Department of Pediatric Ophthalmology and Strabismus, Dr. Shroff’s Charity Eye Hospital, Daryaganj, New Delhi, India; 2 Department of Cornea and Anterior Segment Services, Dr. Shroff’s Charity Eye Hospital, Daryaganj, New Delhi, India

## Abstract

**Objective:** To describe a case of surgically induced scleral necrosis in Treacher Collins syndrome after strabismus surgery.

**Methods:** A 19-year-old girl underwent bilateral squint surgery. Two weeks postoperatively, she presented with subconjunctival abscess in the left eye. The surrounding conjunctiva was markedly inflamed with raised edges. Surgical debridement, microbiological evaluation and medical management were started immediately. Screening for autoimmune and vasculitic conditions did not provide any positive results.

**Results:** On subsequent follow-up, conjunctival retraction and an area of scleral necrosis with thinning was noted. Significant healing with antibiotics and steroids was noted within one week. The integrity of the globe was well maintained and no further procedure for tectonic support was performed.

**Conclusion:** Surgically induced scleral necrosis can be immune-mediated or following surgical site infection. Pre-existing scleral thinning due to neuroectodermal apoptosis in Treacher Collins syndrome remains a possible explanation for the accelerated necrotising scleritis in our case.

## Introduction

Surgically induced scleral necrosis (SINS) is a rare but devastating ocular complication. It is usually reported after cataract and pterygium surgery [1], [2], [3]. However, cases of SINS following strabismus surgery, trabeculectomy and vitrectomy have also been noted [4], [5], [6], [7]. Wegener’s granulomatosis, rheumatoid arthritis, thyroid eye disease, gout and diabetes are the common systemic associations of this condition [8], [9], [10], [11]. However, the diagnostic dilemma has always been between diagnosing a predisposing infection or an immune-mediated cause which leads to scleral melt. It has been hypothesized that SINS results from a hypersensitivity reaction against an antigen revealed or altered due to multiple surgeries. Most of the cases heal with prompt treatment with steroids and control of infection. Surgical debridement, tectonic support with scleral patch graft and corneal grafting have been performed in a few cases refractory to medical management. Treacher Collins syndrome (TCS) is a rare congenital disorder of craniofacial development that arises as the result of mutations in the TCOF1 gene [12]. It is a bilateral anomaly, with zygomatic, temporoaural, and mandibular dysplasia. We report a case of SINS in TCS following strabismus surgery.

## Case description

**Figure 1:**
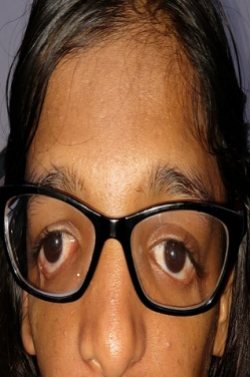
The facial dysmorphic features of Treacher Collins syndrome

A 19-year-old girl presented with large-angle V-pattern exotropia. She was a known case of Treachery Collins syndrome. On examination, she had downward slanting palpebral fissures, micrognathia, microtia, maxillary hypoplasia and zygomatic hypoplasia (Figure 1 (Fig. 1)). She had undergone right lower lid coloboma repair 4 years prior.

**Figure 2:**
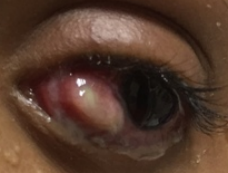
Ruptured subconjunctival abscess

On examination, best-corrected visual acuity was 6/18 in the right eye and 6/12 in the left eye. The refraction was found to be –3.5 dioptres cylinder (DC) at 180 degrees in the right eye and –1.75 DC at 180 degrees in the left eye. The patient had 70 prism dioptres (PD) of exotropia with a V pattern. Ocular motility was full and free on ductions and versions. Magnetic resonance imaging (MRI) orbit did not show any abnormalities in the extraocular muscles position. We decided for a staged approach for ocular alignment. However, in view of the orbital appearance, to attain maximum cosmesis, undercorrection was targeted. We believed that targeting orthotropia or a small esotropia in presence of such external appearance may lead to more cosmetic disfigurement. Both eyes lateral rectus recession (LR) (8 mm) with full tendon width upshift and right eye medial rectus (MR) resection of 6.5 mm was done as the first stage. We used 6-0 vicryl suture (polyglactin 910) for securing the recti, whereas 8-0 vicryl was used for conjunctival closure. Topical povidone iodine 5% was used before and after surgery to maintain asepsis. Postoperatively, the patient was prescribed topical steroids, antibiotics and lubricants as per hospital protocol. She had a residual exotropia of 30 PD in the subsequent follow-up visits. Second-stage surgery was performed 6 months later. Right eye 3 mm LR re-recession and 2 mm MR re-resection with left eye MR resection 6 mm was done. A week following the second surgery, the patient presented with congestion, chemosis over the medial rectus and mild purulent discharge in the left eye. She was started on oral antibiotics. Topical steroids and antibiotics were continued in both eyes. Five days later, she returned to the clinic with a ruptured subconjunctival abscess in the left eye, just posterior to the medial rectus insertion (Figure 2 (Fig. 2)).

There were no signs of intraocular inflammation in any eye. Urgent exploration and debridement were performed on the same day. Intraoperatively, pus was found over the 6-0 vicryl suture placed on the MR muscle. Extensive debridement and betadine-antibiotic wash were done. The suture was not removed in view of further slippage of muscle. Mucopurulent material from the subconjunctival abscess was sent for culture. The culture grew *Staphylococcus aureus* that was sensitive to ceftazidime and cefotaxim. The patient was started on intravenous cefotaxim for three days followed by oral antibiotics for five days. The laboratory evaluation revealed a normal complete blood count, rapid plasma reagin, anti-neutrophil cytoplasmic antibody, serum lysozyme, angiotensin-converting enzyme, hepatitis C panel, erythrocyte sedimentation rate, antinuclear antibody and rheumatoid factor.

**Figure 3:**
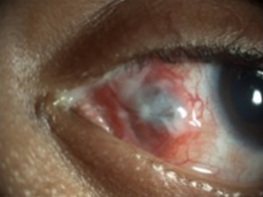
Resolving subconjunctival abscess with scleral necrosis and necrosed medial rectus

Topical steroids were discontinued. On subsequent follow-up, there was a reduction of chemosis, congestion and discharge. On slit-lamp examination, medial rectus posterior to the insertion appeared necrosed. The underlying sclera also appeared thinned (Figure 3 (Fig. 3)).

We presumed post-surgical necrotising scleritis as the possible diagnosis and the patient was then started on oral prednisolone (1 mg/kg body weight). Topical steroids were then re-started to the existing treatment regimen. Oral doxycycline was also initiated to accelerate healing.

**Figure 4:**
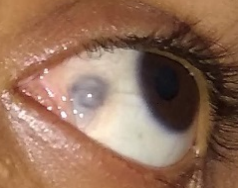
Resolved inflammation with formation of staphyloma

Over the ensuing week, there was significant resolution of infection, though there was uveal show with vascularization. The cornea, anterior chamber, iris, vitreous, and retina remained stable with no signs of intraocular inflammation. Further follow-up showed no further thinning of the sclera or necrosis of the MR. Oral steroids were then stopped. There was residual exotropia of 20 PD. However, in view of developing vascularization and stable scleral integrity, any patch graft for tectonic support was not required further. In the next two months follow-up, the patient remained in good health with stable alignment and vision. The active inflammation reduced with formation of a localized staphyloma (Figure 4 (Fig. 4)).

## Discussion

Treacher Collins syndrome or mandibulofacial dysostosis is characterized by multiple craniofacial abnormalities resulting from incomplete development of the first and second branchial arch [12]. Involvement of the eye adnexa and the orbit are usually associated with craniofacial deformity. The ocular and visual sequalae are a result of the malformation rather than direct involvement of the eye. A common ocular manifestation is strabismus, in the form of esotropia or exotropia, Duane syndrome or cranial nerve palsies [12], [13]. Early management of these cases are necessary to prevent vision loss due to amblyopia. Many children present late with large angle strabismus which requires surgical correction. We performed two-staged surgery for correction of exotropia in this patient. The second stage was re-recession and re-resection to tackle residual exotropia. SINS following strabismus surgery has been reported by few authors [4], [5], [10], [11]. A history of multiple surgeries in the same eye remains another important predisposing factor [1]. We also noted scleral necrosis following the second surgery. However, the site of necrosis was left medial rectus, which had not been operated before in the first stage. Donoghue et al. [2] report predilection of area anterior to lateral rectus insertion for SINS following strabismus surgery. In our case, SINS was noted just posterior to medial rectus insertion. Akbari et al. [14] and Hyuang et al. [10] also reported a case of SINS in the nasal aspect following strabismus surgery. The latency period between surgery and diagnosis of SINS ranged from five days to 51 years [15]. We noted scleral necrosis two weeks following the second strabismus surgery. 

Though there are multiple reports on postoperative scleral melt, the underlying mechanism of this entity is still controversial. It is considered by many that SINS is an inflammatory response to infectious as well as non-infectious predispositions. It is believed that infection can trigger the release of inflammatory mediators that leads to scleral necrosis [1], [4], [7], [9], [16]. The other theory is that any ischemic insult sensitizes the immune system, resulting in a delayed hypersensitivity response, and that this may lead to secondary infection [17]. In our case, the initial presentation mimicked an infective abscess which mislead the suspicion of SINS. In view of positive microbiological evaluation, and good response to systemic and topical antibiotics, we presume infection leading to subsequent scleral necrosis. However, further treatment with oral and topical steroids prevented further scleral melt and loss of ocular integrity. Any tectonic support in the form of a patch graft was thus not required in our case.

Thus, we consider ischemia and infection both as the inciting stimulus. Vascular disruption following squint surgery along with superadded infection triggered the immune-related process resulting in scleral necrosis.

Polyglactin suture has been described to increase the local inflammation and enhance the antigen presentation in eyes developing SINS following pars plana vitrectomy of patients [18]. In this case on surgical exploration, purulent discharge was found over 6-0 vicryl suture placed on medial rectus muscle. This prompts us to consider suture-related infection as the initial infective event leading to scleral necrosis later.

Gregory et al. [19] describe SINS as the result of the products of inflammation and lytic enzymes released by the surrounding conjunctival tissue in the absence of infection or immune related association.

In this case, our observations favoring infective mechanism of SINS are absence of any clinical or serological signs of underlying immunological disorder, and early presentation after surgery.

We also postulate that some inherent embryological abnormality could explain the aggravated necrosis following surgery in these children with TCS. At the cellular level, craniofacial anomalies associated with TCS are believed to arise from abnormalities in neural crest cell proliferation and migration due to extensive neuroepithelial apoptosis [20]. Embryologically human sclera is also a derivative of the neural crest cells. Possibly, these defects in neural crest cells could lead to inherent thin sclera and altered collagen in TCS. Although the initial inciting event was subconjunctival abscess, thin sclera in these eyes predisposed towards aggravated necrosis. However, measurement of scleral thickness by anterior segment optical coherence tomography could have further confirmed this.

## Conclusion

Necrotizing scleritis post strabismus surgery can either be associated with a predisposing systemic condition or an infective source. Careful evaluation and prompt management is always desirable to prevent devastating sequalae. Not all cases will necessitate scleral or corneal patch graft if medical intervention is commenced at the correct time.

## Notes

### Competing interests

The authors declare that they have no competing interests.

### Informed Consent

Written informed consent for publication of this case report and the images was obtained from the patient.
